# Cell softening in malignant progression of human lung cancer cells by activation of receptor tyrosine kinase AXL

**DOI:** 10.1038/s41598-017-18120-4

**Published:** 2017-12-19

**Authors:** Keisuke Iida, Ryo Sakai, Shota Yokoyama, Naritaka Kobayashi, Shodai Togo, Hiroshi Y. Yoshikawa, Anchalee Rawangkan, Kozue Namiki, Masami Suganuma

**Affiliations:** 1 Graduate School of Science and Engineering, Saitama University, Sakura-ku, Saitama, 338-8570 Japan; 2 Research Institute for Clinical Oncology, Saitama Cancer Center, Kitaadachi-gun, Saitama, 362-0806 Japan; 3 Department of Chemistry, Saitama University, Sakura-ku, Saitama, 338-8570 Japan; 4 Present Address: Molecular Chirality Research Center and Department of Chemistry, Graduate School of Science, Chiba University, Inage, Chiba, 263-8522 Japan

## Abstract

To study the role of cell softening in malignant progression, Transwell assay and atomic force microscope were used to classify six human non-small cell lung cancer cell lines into two groups: a high motility-low stiffness (HMLS) group and a low motility-high stiffness (LMHS) group. We found a significant role of activity of the AXL receptor tyrosine kinase, which belongs to the TAM (Tyro3, AXL, Mer) family, in the stimulation of motility and cell softening. HMLS cells expressed higher AXL levels than LMHS cells and contained phosphorylated AXL. H1703 LMHS cells transfected with exogenous *AXL* exhibited increased motility and decreased stiffness, with low levels of actin stress fibre formation. Conversely, the AXL-specific inhibitor R428 and *AXL*-targeting siRNA reduced motility and increased stiffness in H1299 HMLS cells. Knockdown of *AXL* stimulated actin stress fibre formation, which inhibited tumour formation in a mouse xenograft model. The Ras/Rac inhibitor SCH 51344, which blocks disruption of actin stress fibres, exerted similar effects to AXL inactivation. We therefore propose that the Ras/Rac pathway operates downstream of AXL. Thus, AXL activation-induced cell softening promotes malignant progression in non-small cell lung cancer and represents a key biophysical property of cancer cells.

## Introduction

Recent advances in biomechanical tools, such as atomic force microscope (AFM), a microfluidic optical stretcher, and magnetic tweezer system, have led to new discoveries in cancer research. Cancer cells exhibit quantitatively different biophysical properties, such as cell stiffness and elasticity, compared with normal cells^1–5^, and these properties are reflected in cell motility, metastasis, and epithelial-mesenchymal transition (EMT)^3,5^. A study of metastatic cells in pleural fluids using AFM reported that cancer cells from lung, breast, and pancreatic cancer patients have significantly lower average values of Young’s moduli, indicating less stiffness (equivalent to smaller elasticity) compared with normal mesothelial cells in the body fluids^6^. Our experiments with AFM revealed that highly metastatic mouse melanoma B16-F10 cells have a two-fold lower cell stiffness compared with low metastatic B16-F1 cells, although their morphologies and growth rates are almost the same^7^. Since highly metastatic cancer cells obtained from various cancer tissues also have less stiffness and smaller elasticity than low metastatic cancer cells, we speculate that the biophysical properties of cancer cells may provide key indications of early diagnosis and prevention of metastasis.

The AXL tyrosine kinase receptor is a member of the TAM (Tyro3, AXL and Mer) family. Previous studies have shown that AXL (also known as UFO) and Mer (also known as Nyk) induce EMT and malignant phenotypes in various cancer cells derived from the lung, breast and pancreas, along with glioma and myeloid leukemia^8–11^. AXL on the cell surface also acts as a transforming gene product in primary human myeloid leukaemia cells^12^. Activation of the AXL tyrosine kinase receptor is induced through various pathways: ligand binding of growth arrest-specific 6 (Gas6)^13^, dimerization with extracellular domains, and auto-phosphorylation of AXL at Y702 and Y703 residues^14^. AXL shows a higher affinity to Gas6 than Mer^9,13^. Highly phosphorylated AXL is frequently found in human NSCLC cell lines and lung cancer tissues, but AXL is not expressed in normal lung tissues^15–17^. High expression of AXL has been associated with poor survival rates along with metastasis in lung, breast, prostate, pancreatic, ovarian and hepatocellular cancers as well as melanomas and gliomas^8–10^.

Previous studies have established a correlation between high expression of AXL and malignant progression and metastasis in lung cancer. In this study, we examined the potential relationship between cell stiffness and motility with AXL in the malignant progression of lung cancer and the underlying mechanism. We found that both motility and cell softening is stimulated by activation of AXL mediated through reducing actin stress fibre formation via the Ras/Rac pathway. This is the first study to reveal the potential mechanism of cell softening in malignant progression.

## Results

### Increased cell motility and reduced cell stiffness of NSCLC

**Figure 1:**
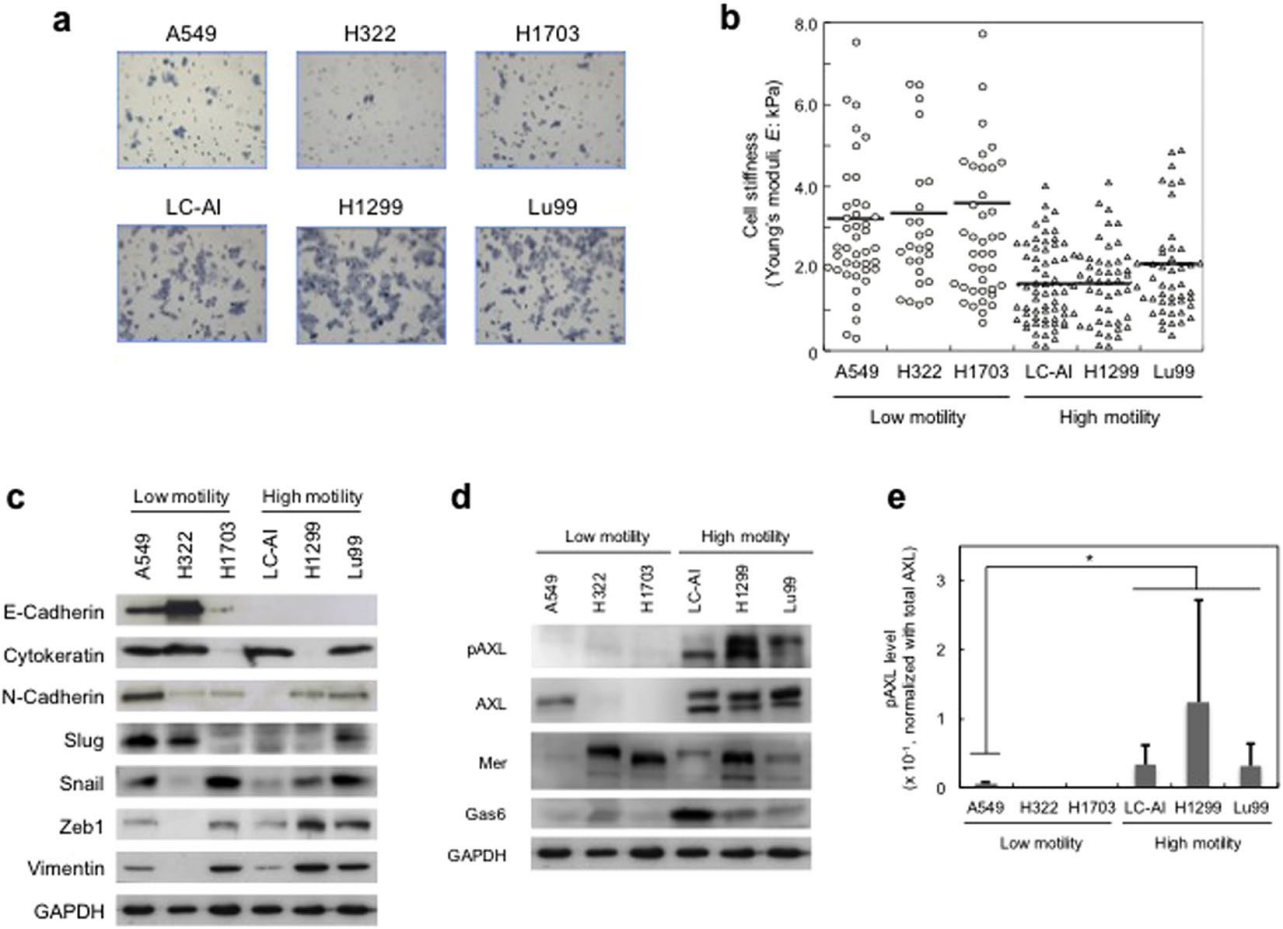
Involvement of AXL tyrosine kinase in cell motility and stiffness of NSCLC. (**a**) Cell motility of six cell lines showing migrated cells attached to the membrane in the presence of 5 µg/ml fibronectin. (**b**) Young’s moduli were determined in six cell lines by AFM. Each dot indicates Young’s modulus of one cell. The low motility cell lines (A549, H322 and H1703) (○) showed wide distribution of Young’s moduli, and the high motility cell lines (LC-AI, H1299 and Lu99) (△) showed narrow distribution of Young’s moduli. Bars indicate median values of Young’s moduli. (**c**) Western blot analysis of EMT-related protein levels in the six cell lines. Western blotting was conducted in at least three independent experiments, and similar results were obtained. (**d**) Western blot analysis of EMT-related receptor tyrosine kinases (AXL and Mer) and Gas6 ligand in the six cell lines. (**e**) Quantitation of pAXL levels in the six cell lines, normalized with total AXL (n = 3) (mean ± s.d.). *p < 0.05.

To examine the role of cell softening (reduced stiffness) in malignant progression and metastasis, we used six human non-small cell lung cancer (NSCLC) cell lines: A549 and H322 from adenocarcinomas, H1703 and RERF-LC-AI (abbreviated to LC-AI) from squamous cell carcinomas, and H1299 and Lu99 from large cell carcinoma for the experiments. The levels of malignancy, metastatic potential, and histological types varied among all the cell lines. We first evaluated the cellular motility of six NSCLC cell lines by Transwell assay and based on the results, we classified the cell lines into two groups. A549, H322 and H1703 cell lines showed fewer than 100 migrated cells (83.5 ± 24.3, 10.5 ± 4.4 and 85.3 ± 25.3, respectively) and were categorized in the low motility group (LMHS), and LC-AI, H1299 and Lu99 cell lines showed more than 200 migrated cells (312.8 ± 111.6, 229.7 ± 70.9 and 257.9 ± 71.2, respectively) and were categorized in the high motility group (HMLS) (Fig. 1a and Table 1). The categorization into low and high motility groups was confirmed by the wound-healing assay results of the six NSCLC cell lines (Table 1 and Fig. S1).

**Table 1:** Motility, cell stiffness and pAXL level in six lung cancer cell lines.

	Motility		Average value of Young’s moduli	pAXL level
	Transwell assay (No. of migrated cells)	Wound-healing assay (μm/24 h)	(*E*: kPa)	(×10^−1^, normalized with total AXL
**Adenocarcinoma**				
A549	83.5 ± 24.3**	10.8 ± 3.1**	2.71 ± 0.14**	0.05 ± 0.02*
H322	10.5 ± 4.4**	9.5 ± 5.3**	3.01 ± 0.31**	0*
**Squamous cell carcinoma**				
H1703	85.3 ± 25.3**	13.0 ± 2.8**	3.18 ± 0.38**	0*
LC-AI	312.8 ± 111.6	43.2 ± 8.8	1.44 ± 0.06	0.34 ± 0.12
**Large cell carcinoma**				
H1299	229.6 ± 70.9	33.0 ± 2.1	1.75 ± 0.04	1.23 ± 0.67
Lu99	257.9 ± 71.2	37.8 ± 8.4	1.66 ± 0.08	0.31 ± 0.14

A549, H322, and H1703 cells belong to LMHS group, and LC-AI, H1299, and Lu99 cells belong to HMLS group. “*” indictes statistical differences between LMHS and HMLS groups. *p < 0.05, **p < 0.01.

We next measured cell stiffness using AFM and found that all three cell lines in the LMHS group showed a wide distribution of Young’s moduli, with high average values of 2.71 ± 0.14, 3.01 ± 0.31 and 3.18 ± 0.38 kPa (Fig. 1b and Table 1). In comparison, the three cell lines in the HMLS group showed a narrow distribution of Young’s moduli, with low average values of 1.44 ± 0.06, 1.75 ± 0.04 and 1.66 ± 0.08 kPa. Histograms of the Young’s moduli in each cell line are shown in Fig. S2. These results indicate that low motility of NSCLC cell lines is strongly associated with high average value of Young’s modulus, which reflects high stiffness, while opposite results are observed in HMLS cells.

### Activation of AXL tyrosine kinase contributes to cell motility of NSCLC

We next evaluated several factors that regulate motility and stiffness of NSCLC cells by performing western blot analysis of EMT-related proteins, such as E-cadherin, cytokeratin, N-cadherin, EMT-related transcription factors and vimentin^18,19^. The results showed E-cadherin expression in three cell lines of the LMHS group at various levels, but no expression was detected in the cells of the HMLS group (Fig. 1c), indicating that the cells of LMSH group possess epithelial phenotypes. Cytokeratin, N-cadherin, EMT-related transcription factors (Slug, Snail and Zeb1) and vimentin showed varying expression among the six cell lines, with no relationship to motility or cell stiffness.

Previous studies established a relationship between high AXL expression and malignancy in lung cancer. We found that AXL levels were significantly higher in the HMLS group compared with the LMHS group (Fig. 1d). In comparison, the expression of Mer, another TAM family tyrosine kinase receptor, was detected in both LMSH and HMLS cell lines, except for A549 cells. Auto-phosphorylation of AXL at Y702 (pAXL), which is required for activation of AXL, was also detected in all three HMLS cell lines (Fig. 1d,e), at levels of 60-fold higher than those of the LMSH group (Fig. 1e and Table 1). These results suggest that activation of AXL may be involved in inducing cell motility and cell softening.

### Downregulation of pAXL level in H1299 cells reduces cell motility and increases cell stiffness

**Figure 2:**
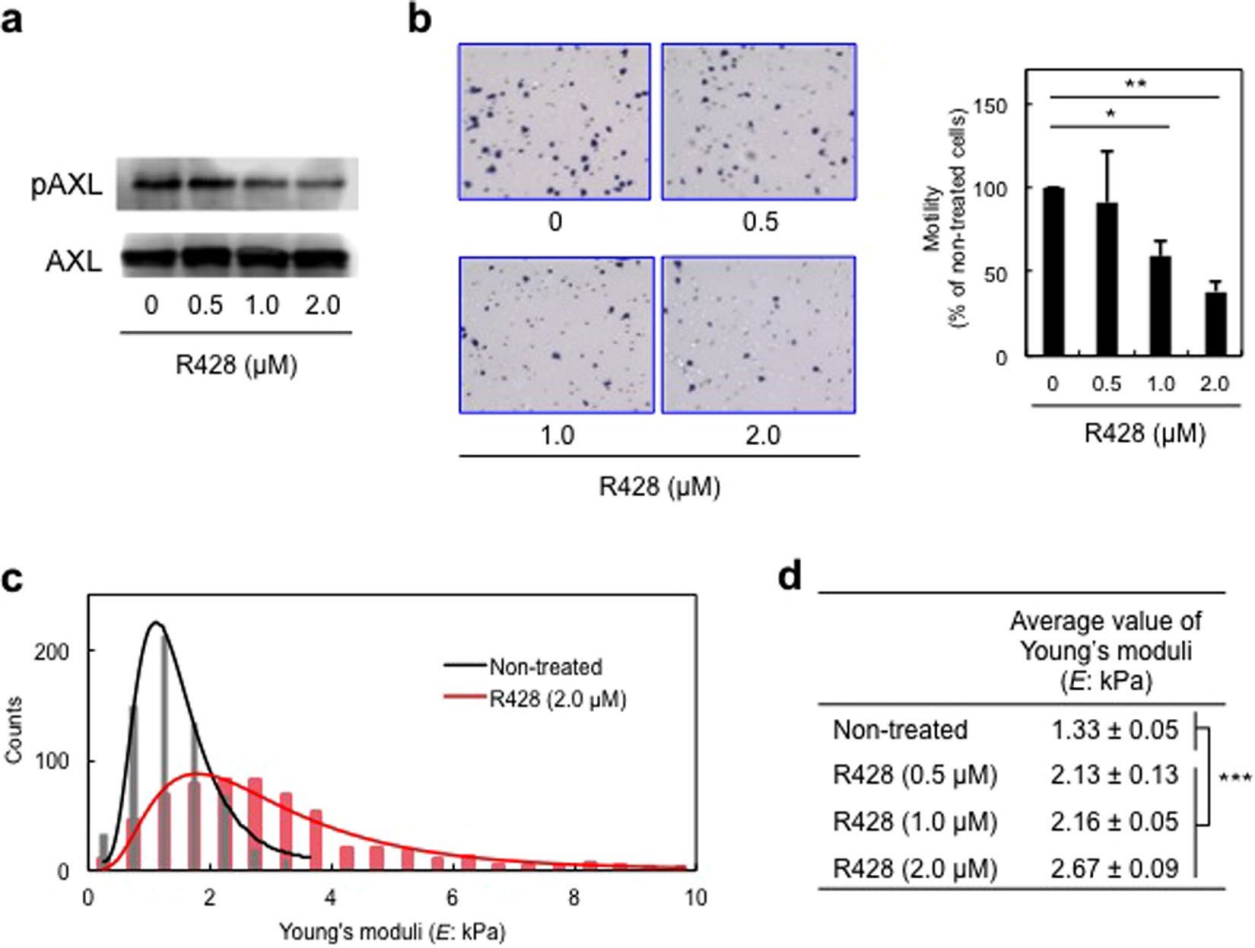
Inhibition of AXL activity in H1299 cells reduces pAXL level and cell motility, and increases cell stiffness. (**a**) Western blot analysis of pAXL level in H1299 cells treated with the indicated concentration of R428 for 1 h. Results were confirmed in three independent experiments. (**b**) Cell motility assays using Transwell assays in H1299 cells treated with the indicated concentration of R428 for 4 h. Left, migrated cells attached to membranes. Right side, average percentages of migrated cells in three independent experiments (mean ± s.d.). Number of migrated cells in non-treated cells was set at 100%. *p < 0.05, **p < 0.01. (**c**) Young’s moduli of 40 cells treated with R428 were determined using AFM. Black line indicates the log-normal fitting curve for non-treated cells, and the red line indicates the curve for R428-treated cells. (**d**) Table shows average values of Young’s moduli for non-treated and R428-treated cells obtained from log-normal fitting curves. ***p < 0.001.

We next examined the relationship between AXL activity and cell motility and stiffness using R428, an inhibitor of AXL and H1299 cells, categorized in the HMLS group with pAXL activation. We confirmed that treatment of H1299 cells with R428 reduced pAXL levels in a dose-dependent manner, with no effects on steady-state AXL protein levels (Fig. 2a), as reported previously^20^. Cell motility assays using the AXL inhibitor showed that treatment with 1.0 and 2.0 μM R428 also inhibited the motility of H1299 cells by 59% and 38%, respectively (Fig. 2b), suggesting a potential role for AXL activation in motility. The treatment of H1299 cells with 2.0 μM R428 increased the average values of Young’s moduli to 2.67 ± 0.09 kPa from 1.33 ± 0.05 kPa of non-treated cells, about 2-fold (Fig. 2c,d). These results suggest that the treated cells became more stiffened than non-treated cells.

**Figure 3:**
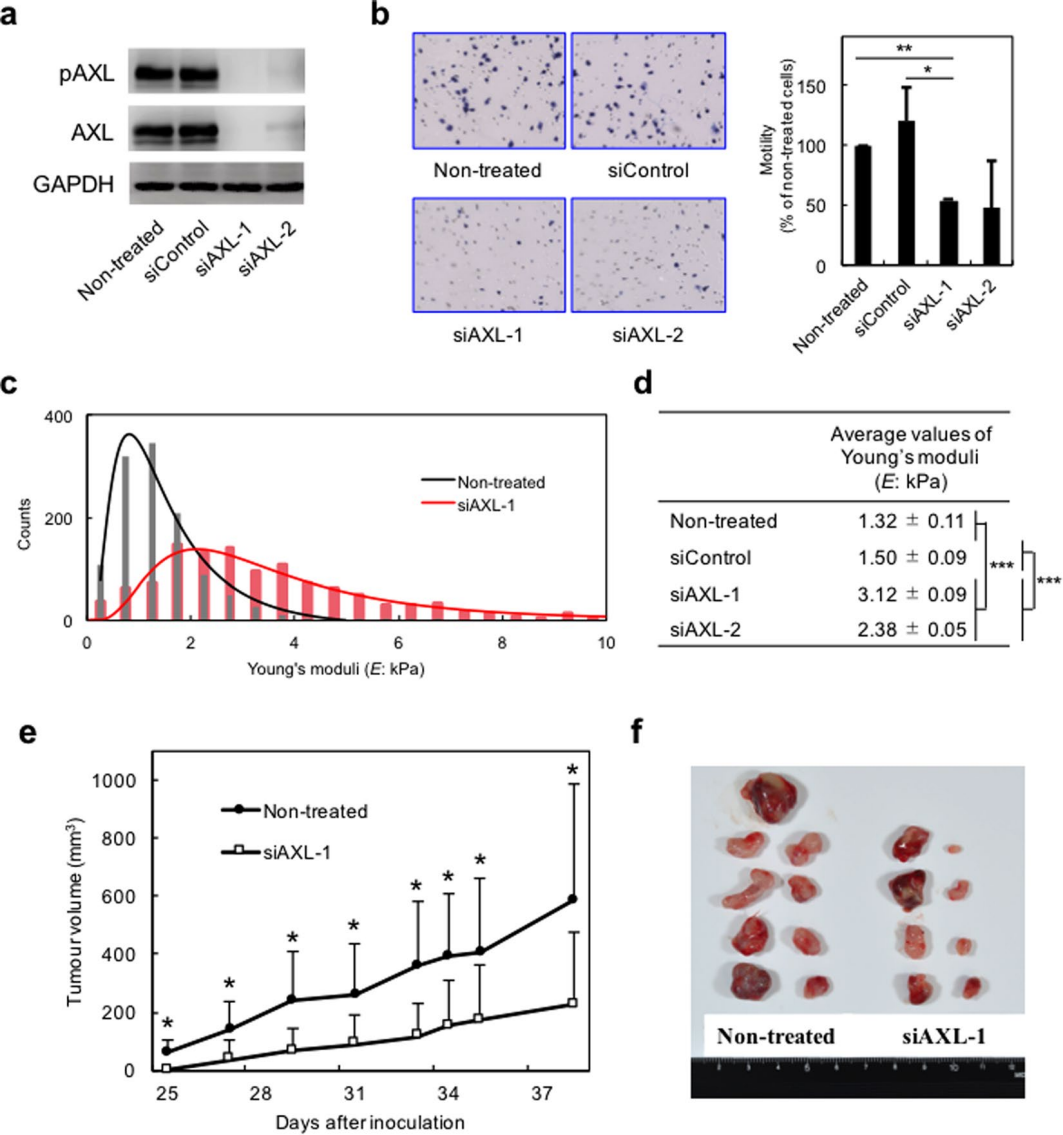
Knockdown of AXL inhibits motility and increases cell stiffness, resulting in inhibition of tumour formation in mouse xenograft model. (**a**) Western blot analysis of H1299 cells treated with medium alone, siControl, siAXL-1 or siAXL-2 for 48 h. The results were confirmed in three independent experiments. (**b**) Cell motility assays in cells transfected with the indicated siRNA. Left, representative images of migrated cells attached to membranes. Right, average percent of migrated cells in three independent experiments. Mean ± s.d. Number of migrated cells in non-treated cells was set as 100%. *p < 0.05, **p < 0.01. (**c**) Young’s moduli of 76–82 cells transfected with siAXL-1 for 48 h were determined using AFM. Black line indicates the log-normal fitting curve for non-treated cells, and the red line indicates the curve for siAXL-1-transfected cells. (**d**) Average values of Young’s moduli were obtained from log-normal fitting curves. ***p < 0.001. (**e**) Tumour volume in a mouse xenograft model of mice implanted with siAXL-1-transfected cells compared with mice with non-treated cells (n = 5 in each group). Tumours were measured every 2 or 3 days. Data represent the average tumour volume and standard deviation. *p < 0.05. (**f**) Image of tumours at the end of the experiment.

To confirm the effects of R428 on H1299 cells, we performed AXL knockdown experiments using siRNA. The transfection of H1299 cells with 10 nM siAXL-1 or 10 nM siAXL-2, two AXL-targeted siRNAs, for 48 h completely inhibited production of AXL and pAXL, whereas siControl transfection did not show any effects (Fig. 3a). In addition, siAXL-1- and siAXL-2-transfected H1299 cells showed significantly reduced motility by 50% and 48%, respectively, compared with that of non-treated cells or siControl-transfected H1299 cells (Fig. 3b). Furthermore, siAXL-1- and siAXL-2-transfected H1299 cells showed average values of Young’s moduli of 3.12 ± 0.09 kPa, and 2.38 ± 0.05 kPa, respectively, which were increased from 1.32 ± 0.11 kPa in the control (Fig. 3c,d). These results indicated that AXL knockdown cells exhibited the high average values of Young’s moduli, i.e., high stiffness similar to that of the LMHS group (A549, H322 and H1703). Together these data clearly showed that inhibition of AXL activity reduces motility, and increases cell stiffness.

### Knocking down of *AXL* gene inhibits tumour development in a mouse xenograft model

To further examine the role of AXL in tumour progression *in vivo*, we used a xenograft mouse model. H1299 cells (5 × 10^6^ cells) were experimentally implanted in subcutaneous tissue of SCID/beige mice. The first tumour from non-treated H1299 cells developed in two mice 25 days later, with an average tumour volume of 582 ± 404 mm^3^ at 38 days later. However, mice implanted with the siAXL-1-transfected H1299 cells developed tumours two days later than mice implanted with non-treated cells, with a smaller average tumour volume (225 ± 248 mm^3^) (Fig. 3e,f). These results confirmed our *in vitro* results and previous evidence indicating the involvement of AXL in tumour growth and progression^10^.

### Knocking down of *AXL* gene increases formation of actin stress fibres

**Figure 4:**
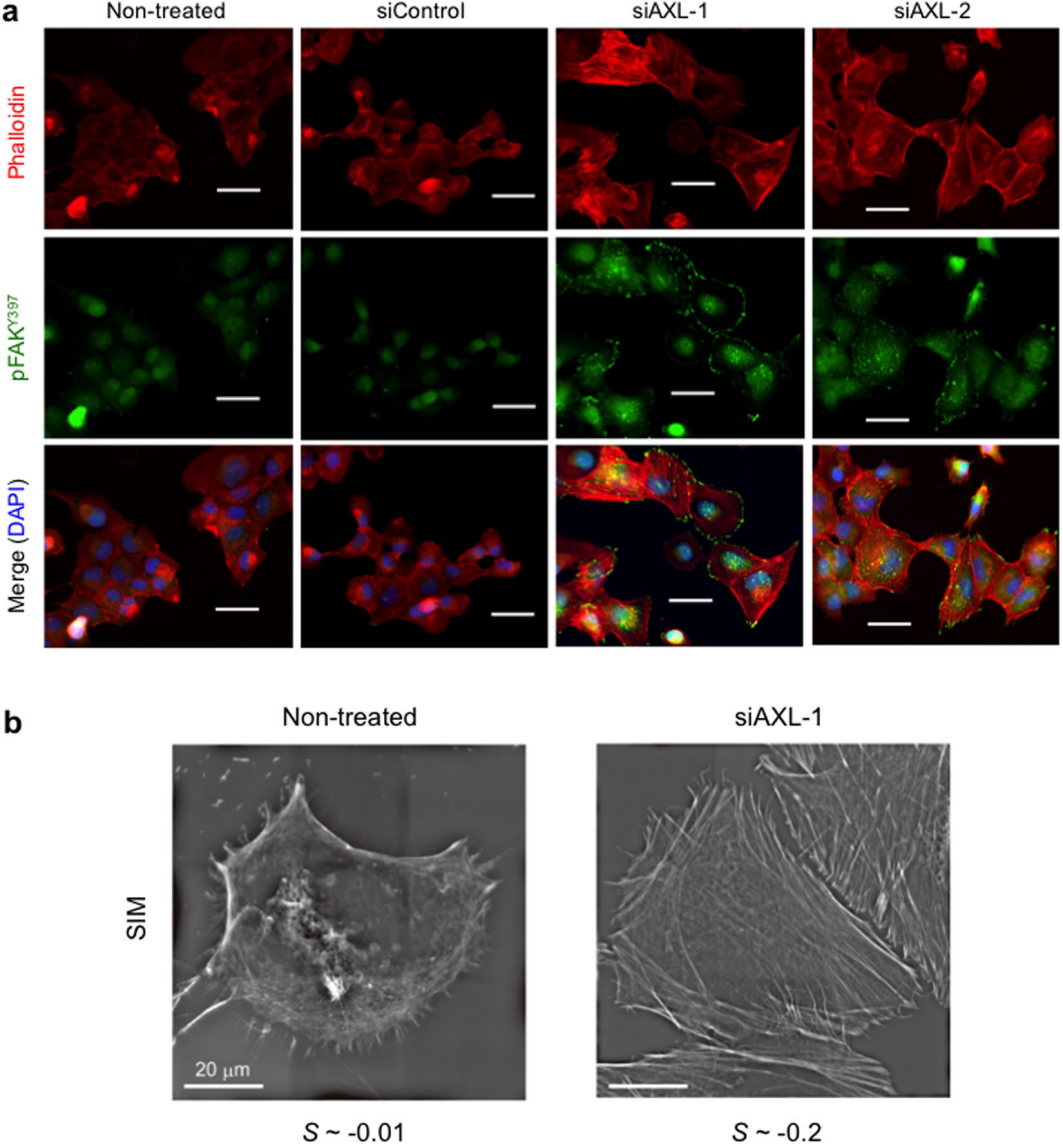
siAXLs increase formation of actin stress fibres. (**a**) Immunohistochemistry of actin stress fibres (red) and pFAK (green) in cells transfected as indicated. DAPI staining (blue) indicates nuclei. (**b**) Actin stress fibres in high resolution in non-treated and siAXL-1-transfected H1299 cells by structures illumination microscopy (SIM). Order parameters of actin stress fibres (*S*) are calculated from each SIM image as shown in Supplementary Information. Similar results were obtained in three independent experiments.

Since remodelling of the actin-cytoskeleton induces the change in cell stiffness^21^, we next studied the formation of actin stress fibres in response to AXL knockdown using phalloidin staining. H1299 cells transfected with siAXL-1 or siAXL-2 showed a strong increase in actin stress fibres, while that with siControl was marginal (Fig. 4a). We also examined the formation of actin stress fibres in both siAXL-1-transfected and non-treated cells using structured illumination microscopy (SIM) with 120 nm resolution (Fig. 4b). The siAXL-1-transfected cells showed long and thick actin stress fibres with directional regularity, while non-treated cells showed short and small fibres without any regularity. The alteration of actin stress fibres was further confirmed with order parameters (*S*) calculated from the SIM images: *S* of siAXL-1-transfected cells was ~0.2 and *S* of non-treated cells was < 0.01 (Fig. 4b). We also found that the transfection of H1299 cells with siAXL-1 and siAXL-2 increased phosphorylated FAK at Y397 (pFAK) at the end of actin stress fibres (Fig. 4a). How knock-down of AXL inceased pFAK needs to be further clarified. Thus, the increase in actin stress fibres is strongly related to AXL level.

### Exogenous expression of *AXL* gene increases cell motility and decreases cell stiffness and actin stress fibres in H1703 cells

**Figure 5:**
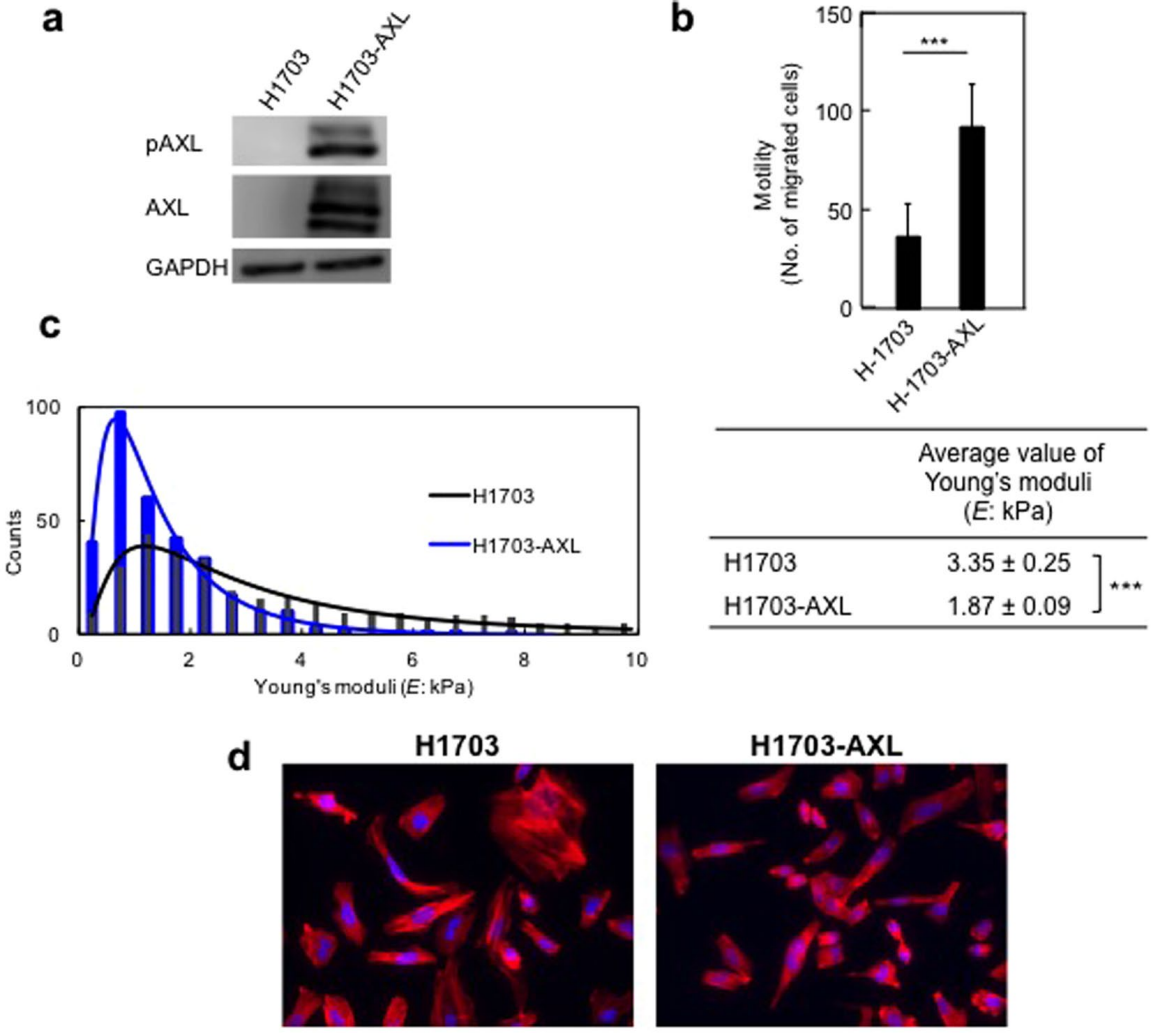
Exogenous expression of *AXL* gene increases motility, and reduces stiffness, formation of actin stress fibres and phosphorylated focal adhesion kinase in H1703 cells. (**a**) Western blot analysis of H1703 cells and the H1703-AXL cell line that stably expresses AXL-GFP. GADPH was used as loading control. (**b**) Cell motility assays of H1703 cells and the H1703-AXL cell line that stably expresses AXL-GFP. Average percentage of migrated cells in three independent experiments. Mean ± s.d. Number of migrated cells in non-treated cells was set as 100%. ***p < 0.001. (**c**) Left, Young’s moduli of 60 H1703 and H1703-AXL cells were determined using AFM. Black line indicates the log-normal fitting curve for H1703 cells, and blue line is the curve for H1703-AXL cells. Right, average values of Young’s moduli were obtained from log-normal fitting curves. ***p < 0.001. (**d**) Actin stress fibres formation (red) in H1703 and H1703-AXL cells. DAPI (blue) indicates nuclei.

Our results have indicated that activation of AXL stimulates cell motility and cell softening (decreases cell stiffness). To further these observations, we used H1703 cells, which do not express AXL (Fig. 1d), for the following experiments. We established H1703-AXL stable cells, which were established and cultured in geneticin. Western blot analysis confirmed that H1703-AXL cells showed marked elevation of AXL-GFP and pAXL-GFP protein levels (Fig. 5a). Transwell assays showed that H1703-AXL cells exhibited a dramatically increased cell motility (about 2.5-fold) compared with H1703 cells (Fig. 5b). The average value of Young’s moduli for H1703-AXL cells was 1.87 ± 0.09 kPa and that for H1703 cells was 3.35 ± 0.25 kPa, indicating a 2.2-fold reduction in cell stiffness from overexpression of the *AXL-GFP* gene (Fig. 5c). Notably, H1703-AXL cells showed a significant decrease in the levels of actin stress fibres compared with parental H1703 cells that do not express AXL (Fig. 5d).

We also examined A549 cells, which express AXL but not pAXL (Fig. 1d). Treatment of A549 cells with Gas6, a ligand of AXL, dose-dependently increased pAXL Y702 levels after 1 h (Fig. S3a), and showed a significant increase in cell motility (about 1.3-fold) compared with non-treated A549 cells (Fig. S3b). The average value of Young’s moduli for Gas6-treated A549 cells was 2.19 ± 0.03 kPa and that for non-treated A549 cells was 2.79 ± 0.07 kPa, indicating that treatment with Gas6 reduced cell stiffness by 1.3-fold (Fig. S3c).

### SCH 51344, a Ras/Rac pathway inhibitor, inhibits motility and increases actin stress fibres and cell stiffness in H1299 and H1703-AXL cells

**Figure 6:**
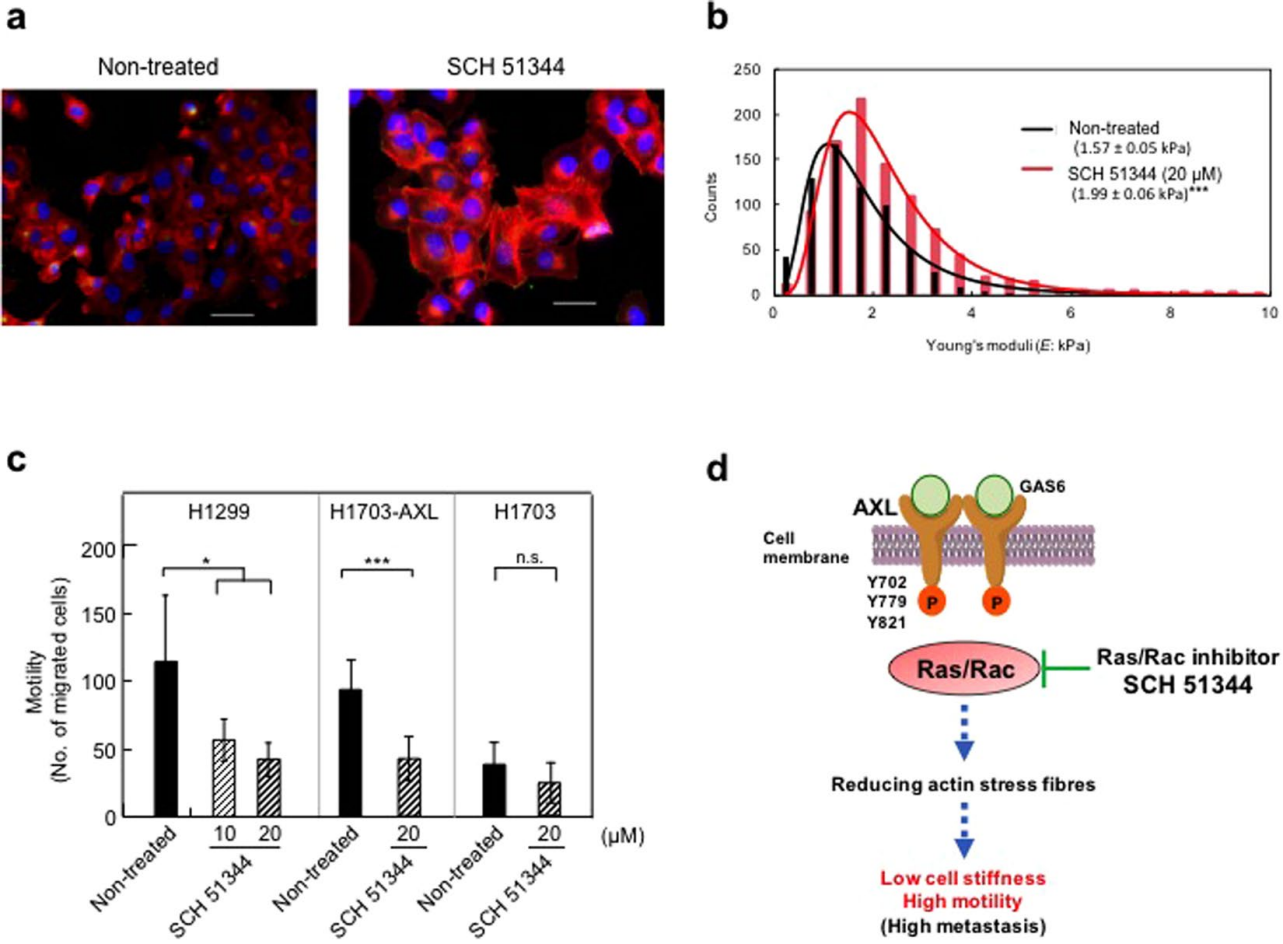
SCH 51344, a Ras/Rac pathway inhibitor, inhibits motility and increases actin stress fibres and cell stiffness in H1299 and H1703-AXL cells. (**a**) Actin stress fibres formation (red) in non-treated H1299 cells and cells treated with 20 μM SCH 51344 for 4 h. (**b**) Young’s moduli of 60 cells in non-treated and SCH51344-treated H1299 cells measured using AFM. Black line indicates the log-normal fitting curve for non-treated H1299 cells, and the red line is the curve for SCH51344-treated H1299 cells. ***p < 0.001. (**c**) Cell motility in H1299 cells and H1703 cells treated with SCH51344. Average number of migrated cells in three independent experiments. Mean ± s.d. *p < 0.05, ***p < 0.001. (**d**) Schematic illustration of the mechanism of stimulation of motility and cell softening by activation of AXL, mediated through the Ras/Rac pathway.

To identify the mechanisms by which AXL reduces actin stress fibre formation, we focused on the Ras/Rac pathway, as the Ras/Rac pathway causes disruption of actin stress fibres during malignant transformation of epithelial cells^22^. SCH 51344, a pyrazolo-qunoline derivative, is an inhibitor of Ras/Rac pathway that blocks Ras/Rac-induced disruption of actin stress fibres and membrane ruffling, resulting in suppression of malignant transformation by oncogenic Ras^23,24^. We found that SCH 51344 treatment of H1299 cells dramatically increased actin stress fibres, with a similar trend as that in siAXL-treated-H1299 cells (Fig. 6a and 4a). SCH 51344 treatment of H1299 cells also increased the average value of Young’s moduli to 1.99 ± 0.06 kPa from 1.57 ± 0.05 kPa of non-treated cells (Fig. 6b), and treatment of H1299 cells with 10 μM and 20 μM SCH 51344 for 4 h significantly decreased cell motility by 46% and 60%, respectively (Fig. 6c). Furthermore, treatment of H1703-AXL cells with 20 μM SCH 51344 reduced motility, but had no impact on H1703 cells. Together these results clearly indicate that AXL stimulates cell motility and softening along with a decrease of actin stress fibres via the Ras/Rac pathway (Fig. 6d).

## Discussion

We previously reported a close relationship between cell motility and cell stiffness in three metastatic B16 melanoma variants (B16-F10, B16-BL6 and B16-F1 cells) and a human gastric cancer cell line (MKN-1), and found that cell stiffness represents the migration potential of cancer cells^7,25^. We also found that treatment of highly motile B16-F10 cells with (−)-epigallocatechin gallate (EGCG), one of the catechins in green tea that inhibits metastasis, inhibited migration and increased cell stiffness by 2-fold as determined by AFM^7^. Similarly, treatment with the carcinogenic factor TNF-α-inducing protein (Tipα) encoded in the genome of *Helicobacter pylori* ^26^ decreased the Young’s modulus of a human adenosquamous gastric cancer cell line (MKN-1) associated with high cell motility and low cell stiffness^25^. Moreover, anticancer drugs, such as taxol, cisplatin and topotecan increase stiffness of various cancer cells^5^. Thus, it is critical to elucidate the underlying molecular mechanisms for this biophysical property of cancer cells to broaden our understanding of cancer and develop cancer treatments and prevention of metastasis.

In this manuscript, we report that the AXL receptor tyrosine kinase is a master regulator of cell softening, one of the biophysical properties in cancer cells, that facilitates malignant progression. Table 2 summarizes the cellular biophysical properties of cells according to high and low AXL activities. Actin cytoskeleton remodelling is reciprocally regulated by two Rho GTPases family, RhoA and Rac^27^. Since SCH 51344, a Ras/Rac inhibitor, clearly inhibited AXL-induced motility and increased cell softening, we conclude that the Rac pathway is downstream of AXL, but not the RhoA pathway, since The Rho-associated protein kinase inhibitor Y-27632 did not inhibit motility of H1299 and H1703-AXL cells (Fig. S5). It is well accepted that AXL activation stimulates several signalling pathways, such as phosphoinositide 3-kinase (PI3K), Akt, MAP kinases and nuclear factor-κB^28^. And Abu-Thuraia *et al*. reported that AXL phosphorylates engulfment and cell motility scaffold proteins, which bind to Dock family guanine nucleotide exchange factors and then stabilizes GTP-binding Rac1 GTPase in breast cancer cells^29^. We think that AXL is a key stimulator of cell softening of cancer cells and cancer progression.

**Table 2:** Cellular effects of activation or inhibition of AXL activity in lung cancer cell lines.

Induced	High AXL in cells of LMHS group	Low AXL in cells of HMLS group
	by transfection of *AXL* gene by AXL ligand, Gas 6	by AXL-targeting siRNA by AXL inhibitor, R428 by Ras/Rac inhibitor, SCH 51344*
pAXL	increased	decreased
Motility	increased	decreased
Tumour formation	n.d.	decreased
Cell stiffness	decreased	increased
Actin stress fibre	decreased	increased

HMLS: high motility-less stiffness; LMHS, low motility-high stiffness.

n.d.: not determined.

*Induced similar phenotypes to low AXL.

A strong correlation between high expression of AXL and metastasis is found in various types of human cancer^8,9^. In NSCLC patients, high expression of both AXL and Gas6 results in poor prognosis with brain metastasis^30^. Phosphoproteomic analyses of 41 NSCLC cell lines and 150 NSCLC tumours revealed that AXL phosphorylates tyrosine residues, similar to other oncogenic kinases such as EGFR, c-Met, and ALK^31^. It is important to note that AXL stimulates the sphere-forming ability, tumourigenicity and expression of CD44^32,33^, and that the elevating the miRNA-processing enzyme Dicer increased the levels of AXL, and reduced the cancer stem-like property in breast cancer^34^. These results support the previous evidence showing that the combination of high expression of AXL and Gas6 protein stimulated metastatic progression and a shorter survival rate of lung adenocarcinoma patients^16,17^.

EMT is an important process in cancer progression and metastasis^18,19^, and AXL is an inducer of EMT-related proteins, such as N-cadherin, Snail, Slug, and vimentin in breast cancer cells^18,19^. And overexpression of AXL is closely associated with expression of EMT-related proteins, such as vimentin, in various cancer models^32^. Transforming growth factor β (TGF-β) is also an EMT-inducing factor^35^, and TGF-β decreases cell stiffness of normal murine mammary gland epithelial cells and reduces RhoA GEFs, LARG and GEF-H1^36^. Silencing of TGF-β inhibits invasion of breast cancer cells, mediated through reduction of the AXL levels^37^. This suggests a strong association between AXL and induction of EMT. However, our experiments with knockdown and exogenous expression of *AXL* did not show any changes in E-cadherin level in NSCLC cells (Fig. S4), probably some other factors are involved in regulation of E-cadherin in NSCLC cells.

Increase of cell stiffness was significantly linked to expression of caveolin-1 in various cancers^38^. Caveolin-1 induces caveolae formation in the plasma membrane and is often downregulated in many tumour-derived or oncogene-transformed cells. Expression of caveolin-1 increases cell stiffness, and upregulates both RhoA activity and pFAK^38^. Knockdown of caveolin-1 by shRNA decreases cell stiffness and stimulates invasion of NIH3T3 cells. Therefore, whether activation of AXL downregulates caveolin-1 expression or whether knockdown of AXL restores caveolin-1 expression in NSCLC cells should be examined in further studies.

Selective small molecules of AXL inhibitors are now attracting attention as new anticancer agents to improve drug resistance^39^. R428 is a potent AXL-specific inhibitor with a molecular weight of 506 Da^20^. It was previously reported that administration of R428 inhibits tumour formation and experimental metastasis in a mouse model of breast cancer metastasis^20^. Our previous report also showed that EGCG in drinking water inhibits spontaneous metastasis of B16-BL6 cells from foot pad into the lungs of C57BL/6 mice^40,41^. Curcumin is a possible agent that increases cell stiffness through the reduction of pAXL associated with low motility of H1299 cells. Kim’s group reported that curcumin induces downregulation of AXL in NSCLC cells, resulting in inhibition of cell proliferation and recovery of chemoresistance^42^. The screening of new agents to increase cell stiffness is now anticipated for the development of non-toxic anticancer agents that are different from the usual tyrosine kinase inhibitors.

In summary, here we report for the first time how the AXL receptor tyrosine kinase regulates motility and cell stiffness in human NSCLC cells. Our results indicate that cell softening induced by activation of AXL is an important biophysical property that enhances cancer progression and metastasis.

## Materials and Methods

### Cell culture and reagents

Human NSCLC cell lines (A549, H322, H1703 and H1299) were obtained from the American Type Culture Collection (VA, USA), and RERF-LC-AI (LC-AI) and Lu99 cells were obtained from Riken Bioresource Center (Tsukuba, Japan). Cells were maintained in RPMI 1640 or MEM (for LC-AI cells) containing 10% foetal bovine serum at 37 °C in 5% CO_2_. Cell line authentication was achieved by genetic profiling using polymorphic short tandem repeat (STR) loci, and results confirmed these cells were the same as the original cell lines. R428, Y-27632 and SCH51344 were purchased from MedChem Express (NJ, USA).

### Transwell assay

Cell motility was determined using a Transwell cell culture chamber (Becton-Dickinson; NJ, USA). Cells (1 × 10^4^) in serum-free RPMI 1640 medium containing 0.1% BSA were added to the upper chamber and incubated for 4 h in the presence of 5 μg/ml fibronectin or 1% FBS in the lower chamber. The cells that migrated to the reverse side of the filter with 8 μm pore were stained with 0.4% trypan blue and counted, as described previously^7^. The data represent an average of three independent experiments that were conducted in duplicate.

### AFM measurement

Cell stiffness was measured by force-map analysis on the nuclear region of a cell using AFM (MFP-3D-Bio-J-AFM, Asylum Research, CA, USA) with a silicon nitride cantilever (TR400PSA, 0.02 N/m, Olympus, Tokyo, Japan), as described previously^7^. Two days after seeding of cells (1–2 × 10^5^/6 cm-dish), 16 force-distance curves in a 4 × 4 matrix in 3 × 3 μm-square on each cell were obtained, and Young’s modulus (*E*: Pa) was calculated from a force-distance curve by fitting with the Hertz model. Stiffness of a cell was calculated as the average value of Young’s moduli from the 10 force-curves represented. After treatment with R428 or siRNA, cells (40–80 in each group) were measured by force-map analysis. Three independent experiments were conducted.

### Western blotting

Whole cell lysates were obtained with lysis buffer containing 20 mM Tris-HCl (pH 8.0), 150 mM NaCl, 1% Triton X-100, 0.1% SDS, 1% sodium deoxycholate, 10 μg/ml aprotinin, 10 μg/ml leupeptin, 1 mM phenylmethanesulfonyl fluoride, 2.5 mM sodium pyrophosphate, 1 mM sodium orthovanadate and 2.5 mM sodium fluoride. Cell lysates were subjected to SDS-PAGE and transferred onto a nitrocellulose membrane. The membranes were blocked with 3% skim milk in 20 mM Tris-HCl (pH 7.6) and 150 mM NaCl with 0.1% Tween 20, and then incubated with the primary antibody overnight at 8 °C. After incubation with horseradish peroxidase-conjugated secondary antibody against rabbit IgG (GE Healthcare, UK) or goat IgG (Santa Cruz, CA, USA), the bound antibody was detected with ImmunoStar LD (Wako Pure Chemical Industries, Ltd., Tokyo, Japan) using a C-DiGit Chemiluminescent Western Blot Scanner (LI-COR Biosciences Inc., NE, USA). The following primary antibodies were used in this study: anti-E-cadherin, anti-N-cadherin, anti-pAXL^Y702^, anti-Slug, and anti-Snail were purchased from Cell Signaling Technology, MA, USA; anti-pan-cytokeratin, anti-Gas6, anti-ZEB1, and anti-vimentin were obtained from Santa Cruz; anti-AXL was purchased from R&D Systems, MN, USA; and anti-GAPDH was from Trevigen, MD, USA. At least three independent experiments were conducted.

### Knockdown of AXL in H1299 cells by siRNA

Two different AXL-targeted siRNAs (s1845 and s1846, called siAXL-1 and siAXL-2, respectively) were purchased from Thermo Fisher Scientific, MA, USA. A control siRNA-A (siControl) was purchased from Santa Cruz Biotechnology. siRNAs at a concentration of 10 nM were transfected into H1299 cells in RPMI 1640 containing 10% FBS using Lipofectamine RNAiMAX Reagent (Thermo Fisher Scientific), according to the manufacturer’s protocol. After 48 h transfection, motility and cell stiffness were measured.

### Tumour xenograft experiment

All animal experiments were performed in accordance with a protocol approved by the Institutional Animal Care and Use Committee of Saitama University. H1299 cells (1 × 10^6^) or siAXL-1-treated H1299 cells (1 × 10^6^) were suspended in 100 µL PBS and injected subcutaneously at two sites per mouse in 5 female SCID/beige mice at 5 weeks of age (Charles River Laboratories, MA, USA). A calliper was used to measure tumour size every 2 or 3 days, and tumour volume (TV) was calculated according to the following formula: TV (cm^3^) = d^2^ × D/2, in which d and D are the shortest and the longest diameters, respectively^43^.

### Phalloidin staining

Cells were fixed with 4% paraformaldehyde containing 0.2% Triton X-100 for 30 min and then incubated with Phalloidin and 4′,6-diamidino-2-phenylindole (DAPI) for 1 h, and observed using a fluorescent microscope (BIOREVO BZ-9000, Keyence, Osaka, Japan).

### Observation of actin stress fibres by SIM

Actin stress fibres in H1299 cells were stained with Alexa Fluor 547-phalloidin (Thermo Fisher Scientific), as previously described^26^. Fluorescent actin images of the fixed samples were acquired using a N-SIM apparatus (Nikon, Tokyo, Japan) with an inverted microscope ECLIPS Ti (Nikon) and a CFI 100 × /1.49 NA Apo TIRF oil immersion objective lens (Nikon). A laser with wavelength of 561 nm was used as an excitation source. Super-resolution SIM images were reconstructed from the captured multiple phases and orientations moiré data using NIS Elements software (Nikon). Images of all cells were reconstructed with images of divided acquisition area of 32.7 × 32.7 µm using a MosaicJ plug-in of ImageJ software (NIH). Obtained SIM images were used for the calculation of order parameter of actin stress fibres. The calculation of order parameter is given in detail in the Supplementary Information.

### Exogenous expression of *AXL-GFP* gene

The cDNA of human AXL (2685 bp) was inserted into the pAcGFP-N1 vector (Clontech Laboratory, CA, USA) at XhoI and HindIII sites, and the resulting vector was transfected into H1703 cells by Lipofectamine 3000 (Thermo Fisher Scientific) according to the manufacturer’s instructions. Cells were cultured in the presence of 0.5 mg/ml geneticin (Thermo Fisher Scientific) for 3 weeks, and then cells with high GFP level were sorted by FACSAria III (BD Biosciences). The resultant H1703-AXL cells were maintained in the presence of 0.1 mg/ml geneticin.

### Statistics

Statistical analyses were performed using nonparametric analysis with Wilcoxon-Mann Whitney for quantification of western blotting, Young’s modulus and tumour volume, or two-sample independent Student’s *t*-test for Transwell assay. The results are expressed as the average ± standard deviation (s.d.). *p* < 0.05 was determined as significant.

## Electronic supplementary material

Supplementary Figures
