# Supplementary material for: Cell softening in malignant progression of human lung cancer cells by activation of receptor tyrosine kinase AXL
Source: Sci Rep. 2017 Dec 19;7:17770. doi: 10.1038/s41598-017-18120-4 (PMC5736582; doi:10.1038/s41598-017-18120-4)
Supplement: Supplementary file 1 — Supplementary Figures [file 41598_2017_18120_MOESM1_ESM.doc]

Supplementary Information

**Cell Softening in Malignant Progression of Human Lung Cancer Cells by Activation of Receptor Tyrosine Kinase AXL**

Keisuke Iida^1,2^, Ryo Sakai^1,2^, Shota Yokoyama^1,2^, Naritaka Kobayashi^1^, Shodai Togo^3^, Hiroshi Y. Yoshikawa^3^, Anchalee Rawangkan^1,2^, Kozue Namiki^1,2^ & Masami Suganuma^1,2^

^1^Graduate School of Science and Engineering, Saitama University, Sakura-ku, Saitama 338-8570, Japan. ^2^Research Institute for Clinical Oncology, Saitama Cancer Center, Kitaadachi-gun Saitama, 362-0806, Japan. ^3^Department of Chemistry, Saitama University, Sakura-ku, Saitama 338-8570, Japan.

Correspondence and requests for materials should be addressed to M.S. (e-mail: masami0306@mail.saitama-u.ac.jp)

**Supplemental Methods**

**Wound-healing assay**

Six NSCLC cell lines were cultured in a 3.5 cm-culture dish for 2 days until confluent. A scratch of about 90 μm in width was made using a 200 μl tip, and cells were cultured for 24 h in serum-free medium. The width of the scratch was measured by taking a photo under a phase contrast microscope (Eclipse Ti-S, Nikon) just after the scratch was made (0 h) and 24 h after incubation. Motility was expressed as the difference of width (μm/24 h) between 0 h and 24 h. The values are averages of three independent experiments conducted in duplicate.

**Calculation of order parameter**

Order parameters (*S* = cos<2*θ*>*)* of actin stress fibres were calculated from the SIM images. *θ* is the angle between each actin fibre in the cell and the long axis of the fitted ellipse. The amount of actin fibres at each orientation was identified by image analysis with a series of elongated Laplace of Gaussian (eLoG) filters. The algorithm uses a series of eLoG filters to create a maximum response image, as described by Zemel et al^1^. First, *n* anisotropic Gaussian of the following forms


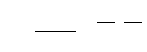


were generated, and each was rotated in steps of π/*n* from 0 to π − π/*n*. In this work, *n* = 12, *σ_x_* = 2 and *σ_y_* = 1 were used. The Gaussian kernels were then convoluted with the Laplacian filter given by


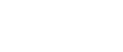
 .

The *n* eLoG kernels were each convoluted with SIM images in which a single cell was clipped out beforehand. This convolution produced *n* response images. The maximum over the *n* response images at each *xy* pixel was used to create a maximum response image. This maximum image was then processed using an intensity threshold to yield the binary mask of the segmented stress fibres. In addition, small round bodies were removed from the image to cut off nonfibrous actin clusters. Finally, the order parameter was determined from the histogram of pixel numbers and multiplied by the corresponding fluorescence intensities (and thus the amount of actin fibres) for each orientation.

**Figure S1 | Motility of six Figure S1 | NSCLC cell lines examined by wound-healing assay.** Photos are representative of wound-healing assays in six NSCLC cells. Red dotted lines indicate the edges of scratch at 0 h and 24 h later.

**Figure S2 | Histograms of Young’s moduli in six NSCLC cells.** Young’s moduli were obtained from 26–62 cells for each cell line. Red curves are log-normal fitting curves.

**Figure S3 | Stimulation of motility and cell softening in A549 cells induced by Gas6.** (a) Increase of pAXL (AXL phosphorylated at Y702) was induced by Gas6. Western blot analysis of pAXL and AXL in A549 cells treated with various concentrations of Gas6 for 1 h. (b) Increase in motility of A549 cells induced by Gas6 in Transwell assay. A549 cells were treated with indicated concentration of Gas6 for 1 h and images show migrated cells attached to the membranes (left side). Average percentages of migrated cells in three independent experiments. Mean ± s.d. (right side). Number of migrated cells in non-treated cells was set as 100%. **p* < 0.05. (c) Average value of Young’s moduli for Gas6-treated cells decreased to a low average value compared with that for non-treated cells. Left, Young’s moduli of 60 cells determined by AFM at 4 h after incubation with either medium alone or Gas6 (500 ng/ml) in serum-free medium. Black line is the log-normal fitting curve for non-treated cells, and the blue line is the curve for Gas6-treated cells. Right, table shows average values of Young’s moduli for non-treated and Gas6-treated cells obtained from log-normal fitting curves. ***p* < 0.01.

**Figure S4 | Alteration of AXL level does not affect E-cadherin and N-cadherin protein levels.** (a) E-cadherin and N-cadherin are not affected by AXL levels in H1299 cells. Western blot analysis of H1299 cells transfected with siAXL-1 and -2 for 48 h. (b) No significant change in E-cadherin and N-cadherin in H1703 cells stably expressing AXL. Western blot analysis of E-cadherin or N-cadherin H1703 cells and H1703 cells overexpressing AXL. (c) Gas 6 treatment does not impact E-cadherin or N-cadherin in A549 cells until 24 h post-treatment. A549 cells were treated with Gas6 (500 ng/ml) for the indicated times and E-cadherin and N-cadherin were evaluated by western blot.

**Figure S5 | Rho-associated kinase inhibitor Y-27362 did not inhibit motility of H1299 and H1703-AXL cells.** Cell motility assays using Transwell assays in H1299 cells and H1703-AXL cells treated with Y-27362 (10 μM) for 4 h. n.s.: not significant.

**Reference**

[1] Zemel, A., Rehfeldt, F., Brown, A. E. X., Discher, D. E., & Safran, S. A. Optimal matrix rigidity for stress-fibre polarization in stem cells. *Nat. Phys.* **6**, 468−473 (2010).
